# Chrysoviruses in *Magnaporthe oryzae*

**DOI:** 10.3390/v10120697

**Published:** 2018-12-08

**Authors:** Hiromitsu Moriyama, Syun-ichi Urayama, Tomoya Higashiura, Tuong Minh Le, Ken Komatsu

**Affiliations:** 1 Laboratory of Molecular and Cellular Biology, Department of Applied Biological Sciences, Tokyo University of Agriculture and Technology, 3-5-8, Saiwaicho, Fuchu, Tokyo 184-8509, Japan; urayama.shunichi.gn@u.tsukuba.ac.jp (S.-I.U.); tomoya.higashiura@gmail.com (T.H.); 2 Laboratory of Plant Pathology, Department of Applied Biological Sciences, Tokyo University of Agriculture and Technology, 3-5-8, Saiwaicho, Fuchu, Tokyo 184-8509, Japan; lmtuong@ctu.edu.vn (T.M.L.); akomatsu@cc.tuat.ac.jp (K.K.); 3 Department of Plant Protection, College of Agriculture & Applied Biology, Can tho University, Can tho city 900000, Vietnam

**Keywords:** Mycovirus, rice blast fungus, *Magnaporthe oryzae.* chrysovirus 1, double-stranded RNA virus, hypovirulence

## Abstract

*Magnaporthe oryzae*, the fungus that causes rice blast, is the most destructive pathogen of rice worldwide. A number of *M. oryzae* mycoviruses have been identified. These include *Magnaporthe oryzae.* viruses 1, 2, and 3 (MoV1, MoV2, and MoV3) belonging to the genus, *Victorivirus*, in the family, *Totiviridae*; *Magnaporthe oryzae.* partitivirus 1 (MoPV1) in the family, *Partitiviridae*; *Magnaporthe oryzae.* chrysovirus 1 strains A and B (MoCV1-A and MoCV1-B) belonging to cluster II of the family, *Chrysoviridae*; a mycovirus related to plant viruses of the family, *Tombusviridae* (*Magnaporthe oryzae.* virus A); and a (+)ssRNA mycovirus closely related to the ourmia-like viruses (*Magnaporthe oryzae.* ourmia-like virus 1). Among these, MoCV1-A and MoCV1-B were the first reported mycoviruses that cause hypovirulence traits in their host fungus, such as impaired growth, altered colony morphology, and reduced pigmentation. Recently we reported that, although MoCV1-A infection generally confers hypovirulence to fungi, it is also a driving force behind the development of physiological diversity, including pathogenic races. Another example of modulated pathogenicity caused by mycovirus infection is that of Alternaria alternata chrysovirus 1 (AaCV1), which is closely related to MoCV1-A. AaCV1 exhibits two contrasting effects: Impaired growth of the host fungus while rendering the host hypervirulent to the plant, through increased production of the host-specific AK-toxin. It is inferred that these mycoviruses might be epigenetic factors that cause changes in the pathogenicity of phytopathogenic fungi.

## 1. Introduction

Similar to animals and plants, fungi are often infected by viruses. In general, viral infections in higher eukaryotes result in easily detectable alterations, such as disease, whereas when a simpler eukaryote, such as a fungus acts as a host, it is often hard to recognize the effects of infection. However, numerous viruses of yeasts and filamentous fungi have been reported to cause epigenetic phenomena [1]. Prions, or “infectious proteins”, are also known to affect fungi [2]. Mycoviruses (viruses that infect fungi) were originally described in diseased *Agaricus bisporus* mushrooms [3], in *Penicillium chrysogenum* [4], and in the brewing yeast, *Saccharomyces cerevisiae* [5]. In fact, yeast killer viruses have been used in fermentation production as bio-controllers. Mycoviruses that infect plant pathogenic fungi were initially discovered in the rice blast fungus, *Magnaporthe oryzae* [6], and have since been found in many other plant pathogenic fungi. The hypovirus CHV1-EP713 that infects the chestnut blight fungus, *Cryphonectria parasitica*, is a famous example of a biological control agent used to attenuate host fungal infection [7]. Mycoviruses have also been found in the human pathogenic fungus, *Aspergillus fumigatus*, since 1990 [8].

In many cases, double-stranded RNA (dsRNA) molecules are either the actual genome of the mycovirus or its replication intermediate. Similar to animals and plants, many fungi have antiviral capabilities, which act through RNA interference mechanisms targeting dsRNA degradation [7]. Therefore, it is of interest to investigate the relationship between mycovirus propagation and host defence mechanisms. In recent years, there have been many reports on the interactions between fungal viruses and RNA interference [9,10,11].

It is plausible that there are more than 1.5 million species of fungi and oomycetes on Earth. Many of these organisms, including phytopathogenic fungi, cause enormous damage to agricultural crops [12]. To date, about 8000 phytopathogenic fungi have been reported in Japan alone and they are the most common type of plant pathogen, over 10 times more frequent than viruses and bacteria [13]. In contrast, many more viruses and bacteria infect humans than fungi. Considering that each phytopathogenic fungus is infected with multiple mycoviruses, the number of newly identified mycoviruses will certainly increase in the future.

To date, three distinct dsRNA viruses have been reported to infect *M. oryzae*, including from the genus, *Victorivirus*, *Magnaporthe oryzae.* virus 1 [14] and *Magnaporthe oryzae.* virus 2 [15], *Magnaporthe oryzae.* virus 3, a mycovirus that belongs to the family, *Partitiviridae* (*Magnaporthe oryzae.* partitivirus 1) [16], and a mycovirus that belongs to cluster II of the family, *Chrysoviridae* *Magnaporthe oryzae.* chrysovirus 1 (MoCV1) [17,18]. Recently, a mycovirus related to plant viruses of the family, *Tombusviridae* (*Magnaporthe oryzae.* virus A) [19], and a mycovirus closely related to ourmia-like viruses (*Magnaporthe oryzae.* ourmia-like virus 1) [20] has also been reported.

In this review, we discuss MoCV1, a mycovirus that causes growth inhibition in the rice blast fungus, *M. oryzae*. We focus on its molecular genetic characteristics, the influence of viral proteins on host cells, and our methodology of investigating physiological activity using a yeast heterologous expression system.

## 2. Effects of Magnaporthe Chrysovirus on the Rice Blast Fungus

**Figure 1:**
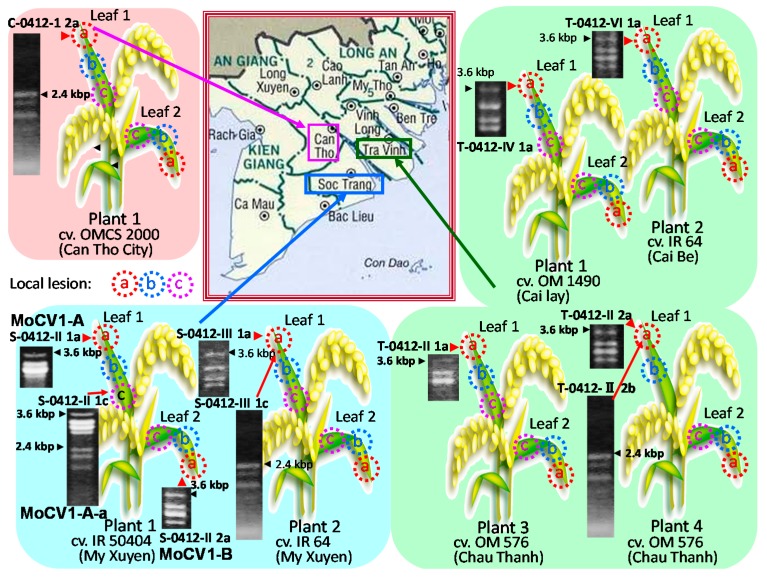
Distribution map of rice blast fungus infected with mycoviruses. Mycoviruses with dsRNA genomes were found in 11 *M. oryzae* isolates from three provinces in the Mekong Delta region of Vietnam. The plant diagrams show the sites where disease lesions were sampled. The agarose gel shows viral dsRNA segments present in the infected *M. oryzae* isolates. Two mycoviruses were identified: MoCV1 had five dsRNA genomic segments of 2.6 kbp to 3.6 kbp, and a partitivirus had three segments of 1.8 kbp to 2.4 kbp. The *M. oryzae* isolates, S-0412-II 1a, S-0412-II 1c, and S-0412-II 2a, were infected with the MoCV1 strains, MOCV1-A, MOCV1-A-a, and MOCV1-B, respectively. Isolate S-0412-II 1c was also infected with the partitivirus.

**Figure 2:**
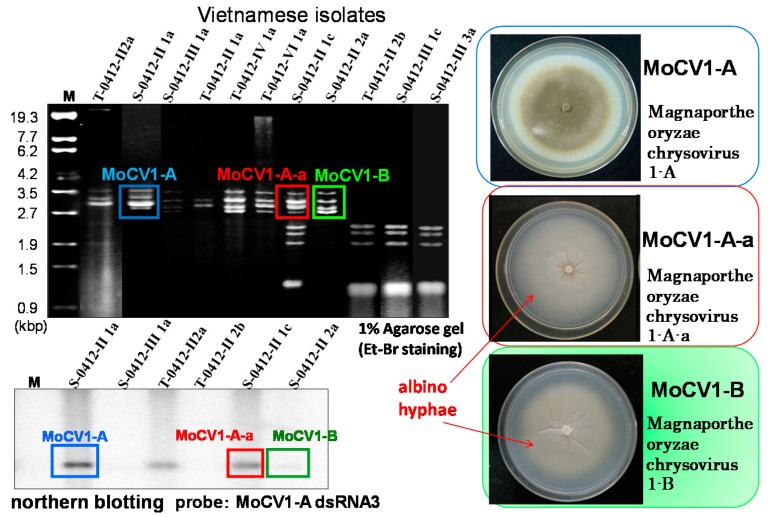
Analysis of mycoviruses isolated from *M. oryzae* in Vietnam. Top left, agarose gel electrophoresis of dsRNAs derived from mycoviruses purified from rice blast isolates. Lower left, northern hybridisation with a cDNA probe derived from MoCV1-A dsRNA3, showing a weak signal for MoCV1-B dsRNA3. Right, fungal flora of the *M. oryzae* isolates infected with MoCV1-A (top), MoCV1-A-a (middle), and MoCV1-B (bottom). These isolates were cultured on PDA medium at 26 °C for 10 days.

**Figure 3:**
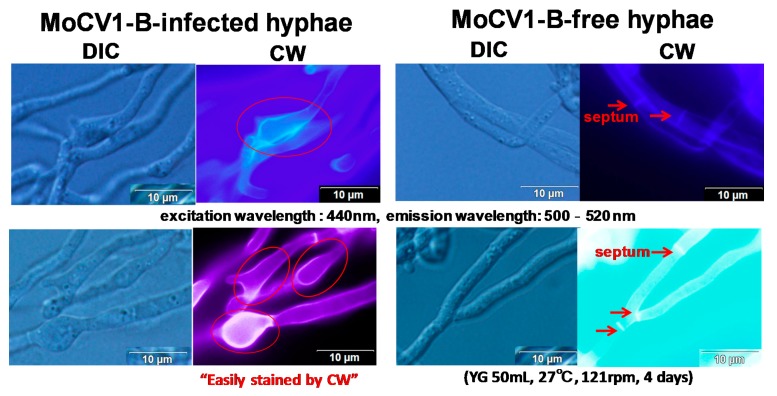
Influence of MoCV1-B on cell wall formation in *M. oryzae* hyphae. Infected and non-infected hyphae were stained with calcofluor-white (Sigma Chemical, St. Louis, MO, USA) and examined at 1000× magnification under a light microscope (Olympus IX71, Tokyo, Japan) with differential interference contrast (DIC) optics. Calcofluor-white (CW) binds strongly to structures containing cellulose and chitin.

The rice blast fungus, *M. oryzae*, is a plant pathogen that causes significant damage to rice production annually worldwide. In Japan, the annual rice yield is almost 9 million tons, and almost 1% is typically lost to infection with rice blast [21]. We first investigated the prevalence of mycoviruses in 58 isolates of *M. oryzae* collected in the Mekong Delta area of Vietnam. We screened for dsRNA in cell extracts using a simple purification method [22] and found dsRNA elements in 11 isolates (Figure 1) [17]. Among these, a virus with a dsRNA genome of 2.6 kbp to 3.6 kbp was nominated as *Magnaporthe oryzae.* chrysovirus 1 (MoCV1). The *M. oryzae* isolates were also infected with a partitivirus whose three dsRNA genomic segments ranged in size from 1.8 kbp to 2.4 kbp. Some isolates were infected with only one of these two mycoviruses while others were infected with both (Figure 2, top left). Among the MoCV1 strains detected in Vietnam, the one that was most stably maintained in liquid medium or on agar plates was nominated MoCV1-A. In another strain nominated MoCV1-B, the content of dsRNA obtained from cells in liquid culture was sometimes lower than that obtained with MoCV1-A (Figure 2) [18]. Since the hyphal morphology of rice blast fungus infected with MoCV1-B showed remarkable white pigmentation on agar medium (Figure 2, right), melanin biosynthesis appears to be suppressed by MoCV1-B infection. Melanin biosynthesis mutants of *M. oryzae* are unable to invade rice leaves due to deficiencies in appressorium formation, and thus have greatly reduced infectivity. Since the fungal melanin biosynthesis pathway is different from the human one, specific compounds, such as sytalone or 1,3,6,8-tetrahydroxynaphthalene, can be used as pesticides for controlling the rice blast fungus [23,24]. Also, no conidial formation was observed in strains infected with MoCV1-B on PDA (Potato dextrose agar) medium [18]. Since *M. oryzae* propagates by spreading conidia in the air, we would expect that the suppression of conidial formation would limit the spread of the rice blast fungus. Furthermore, the cell wall of a MoCV1-B infected strain, as observed under the microscope, was loose and enlarged [18], and staining with calcofluor-white showed that the cell wall was damaged (Figure 3).

## 3. Molecular Properties of MoCV1-A and MoCV1-B

**Figure 4:**
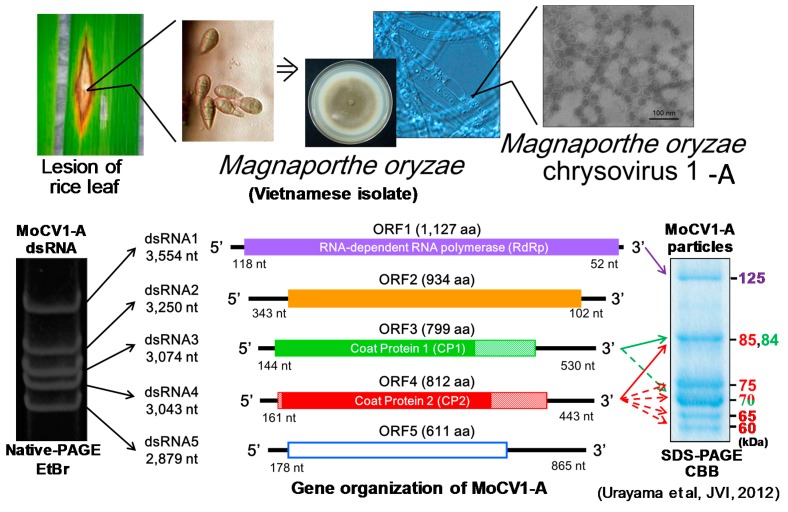
Isolation and analysis of mycovirus MoCV1-A from the rice blast fungus, *M. oryzae*. Upper panels, process of isolation and purification of the mycovirus. Lower left, dsRNA genomic segments extracted from purified MoCV1-A virus particles were subjected to 5% (*w*/*v*) native PAGE. Lower middle, open reading frames (ORFs) within each of the five genomic segments. Lower right, MoCV1-A viral proteins were separated by denaturing PAGE.

The genome of MoCV1-A has five dsRNA segments (dsRNAs 1–5), and dsRNA1 encodes an RNA-dependent RNA polymerase (RdRp) and dsRNA3 and dsRNA4 each encode a separate coat protein (CP1 and CP2) (Figure 4) [25]. This RdRp has ca. 30% similarity to the RdRps of *Helminthosporium victoriae* virus 145S (HvV145S) and *Penicillium chrysogenum* virus (PcV), which belong to the family, *Chrysoviridae*. Therefore, we classified MoCV1-A in the same family. MoCV1-B dsRNAs 1–4 showed ca. 75% identity with their MoCV1-A counterparts, whereas dsRNA5 had 96% identity between MoCV1-A and MoCV1-B. This suggested that dsRNA5 might have migrated as a satellite RNA between strains of MoCV1 located in the Soc Trang province of Vietnam. Whilst subculturing *M. oryzae* isolate, S-0412-II 2a, which was infected with MoCV1-B, we discovered a MoCV1-B derivative strain lacking a dsRNA5 segment. This derivative strain also formed viral particles that could be released from the fungal cells, suggesting that dsRNA5 is not essential for viral maintenance [18]. The *M. oryzae* isolate, S-0412-II 1c, was co-infected with both MoCV1 and the partitivirus whose genomic segments were 2.4, 2.2, and 1.8 kbp in size. This MoCV1 strain was named MoCV1-A-a because its five dsRNA genomic segments showed more than 99% identity with the counterpart dsRNAs of MoCV1-A.

Initially, the MoCV1-related viruses, MoCV1-A, MoCV1-A-a, and MoCV1-B, were discovered in rice blast isolates from Vietnam. We then used reverse transcription-PCR and a loop-mediated isothermal amplification method that was developed for the specific detection of these mycoviruses, and discovered a MoCV1-related virus (MoCV1-D) in rice blast isolates from Japan [26]. MoCV1-D has five dsRNAs as its genome and dsRNAs 1–4 showed 75%–81% identity with the corresponding dsRNAs from MoCV1-A and MoCV1-B. Conversely MoCV1-D dsRNA5 possessed relatively low identity (63%) and was dispensable for virus propagation, as was the case with MoCV1-B dsRNA5. Whereas the Vietnamese MoCV1 strains were sometimes found together with a partitivirus in the same *M. oryzae* isolates, MoCV1-D was found in combination with a victorivirus, MoV2, and some different partitiviruses. Recently, a rice blast fungus isolated in Hubei, China was found to be co-infected with a victorivirus (MoV3) and a chrysovirus that was designated MoCV1-C; however, MoCV1-C has not yet been sequenced [27].

The family, *Chrysoviridae*, includes a single genus with two large distinct clusters. Cluster I contains members of the genus, *Chrysovirus*, and related unclassified viruses with three genomic segments, while cluster II comprises related, unclassified viruses with four or five genomic segments [28]. Phylogenetic analysis revealed that MoCV1-A and MoCV1-B are members of cluster II, together with the following viruses: Botryosphaeria dothidea chrysovirus 1 (BdCV1) [29], Penicillium janczewski chrysoviruses 1 and 2 (PjCV1, PjCV2) [30], Aspergillus mycovirus 1816 (AmV1816) [31], Agaricus bisporus virus 1 (AbV1) [32], Fusarium oxysporum f. sp. mycovirus 1 (FodV1) [33,34,35], Fusarium graminearum mycovirus-China 9 (FgV-Ch-9) [36], Tolypocladium cylindrosporum viruses 1 and 2 (TcV1, TcV2) [37], and Alternaria alternata chrysovirus 1 (AaCV1) [38]. MoCV1-A and AaCV1 are reported to affect fungal pathogenicity [37,39]. FgV-ch9 and Aspergillus thermomutatus chrysovirus 1 (AthCV1) are also reported to attenuate the growth of their host fungi [40,41].

## 4. Virus Particles Containing dsRNAs and Multiform Structural Proteins

Purification of the MoCV1-A and MoCV1-B virus particles was performed using standard methods without solvents to avoid denaturation of viral structural proteins. We found that the buoyant densities of the particles depended on the sizes of the packaged dsRNA segments within them, suggesting that the five dsRNA segments were packaged separately in individual virus particles [17]. Conserved sequences present in the 5’-(GCAAAAAAGAGAAUAAAGC–UUC UCCUUUUUGCA) and 3’-(AAGUACC) terminal regions of each dsRNA may include common packaging signals, replication sites, or ribosomal entry sites.

We extracted and purified MoCV1-A particles from a 14-day-old liquid culture of mycelia. Coomassie Blue staining of SDS-PAGE gels revealed that the particles contained four major proteins 125 kDa, 70 kDa, 65 kDa, and 60 kDa in size [25]. Edman degradation analysis showed that the N-terminus of the 125 kDa protein was blocked, since no phenylthiohydantoin (PTH)-amino acid derivatives were observed. The protein was purified, subjected to trypsin digestion, and analyzed by HPLC. A tryptic peptide in the chromatographic peaks was subjected to Edman degradation, and its sequence matched the amino acid sequence of the protein encoded by ORF1 (the open reading frame of dsRNA1). The 70 kDa protein was also subjected to Edman degradation and was proven to be encoded by ORF4, although it lacked 14 amino acids in the N-terminal region. This suggests that these amino acids are cleaved by post translational modification. The 65 kDa protein was confirmed to be a derivative of the 70 kDa protein following immunoblotting using an antiserum to the ORF4 protein [25].

**Figure 5:**
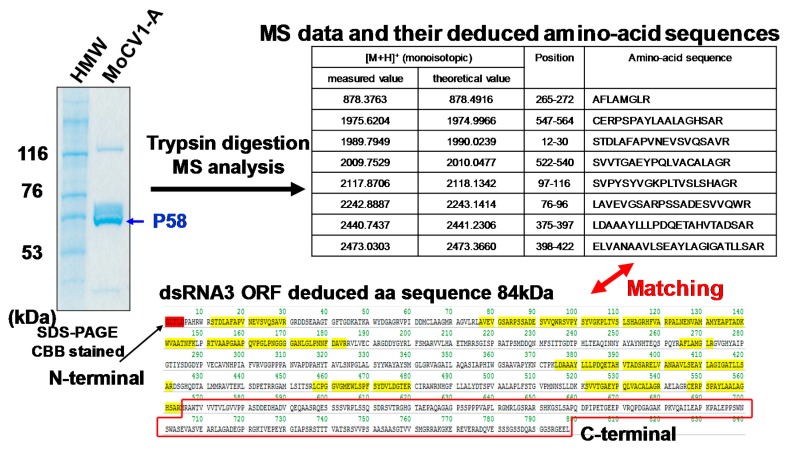
Determination of the C-terminal residue of the MoCV1-A 60 kDa protein (labeled P58 in the figure). The C-terminus of P58 is a truncated version of the ORF3 protein missing 200 amino acids. Mass spectrometry of tryptic peptides derived from P58 identified peptides specific to the deduced amino acid sequence of ORF3, but no peptide sequence mapped to the C terminus of the deduced amino acid sequence of ORF3 (M565 to L799).

The 60 kDa protein was also subjected to Edman degradation and was identified as a partially degraded form of the ORF3 protein. Its N-terminal sequence was consistent with the N-terminus encoded by dsRNA3, however its apparent molecular mass (*Mr*) was smaller than the deduced *Mr* of the ORF3 protein (799 aa, 84 kDa). Mass spectrometry of tryptic peptides revealed that the C-terminus of the deduced ORF3 protein (M565 to L799) was absent from the 60 kDa protein (Figure 5). Then, MALDI-TOF MS (Matrix Assisted Laser Desorption/Ionization-Time of Flight Mass Spectrometry) was performed to determine the exact *Mr* of the 60 kDa protein, and a resultant ion signal was observed at *m*/*z* 62,559. The *Mr* of the deduced amino acid sequence is 62,530 for residues G1 to V590, and 62,658 for residues G1 to Q591. Together, the results suggest that the C-terminal end of the 60 kDa protein might be located around residues M565 to Q591 of the ORF3 protein [25].

**Figure 6:**
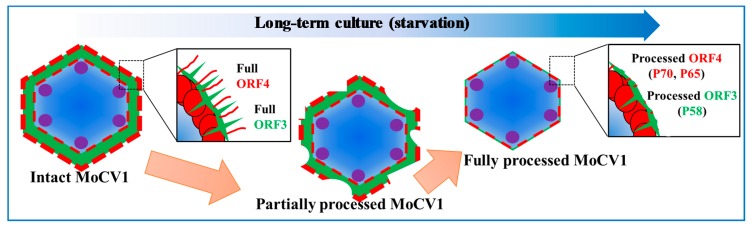
Model for partial degradation of the ORF3 and ORF4 proteins. In the early stage of culture, the MoCV1-A viral particles contained full-length ORF3 and ORF4 proteins. After long-term culture and nutrient depletion, the viral particles were composed of partially degraded ORF3 and ORF4 proteins.

To investigate whether the full-size proteins are components of MoCV1-A particles, we purified virus particles from fresh mycelia that had been grown in a fermenter for two days. The purified isometric virus particles had buoyant densities of 1.38 g cm^−3^ to 1.40 g cm^−3^ in CsCl and diameters of about 35 nm. Anti-ORF3 antiserum detected an 84 kDa protein corresponding to the full-size protein encoded by ORF3, in addition to 75, 70, 66, and 60 kDa proteins. Anti-ORF4 antiserum detected an 85 kDa protein corresponding to the full-size ORF4 protein as well as a 70 kDa protein. These results indicated that full-size ORF3 and ORF4 proteins might be components of MoCV1 particles as coat protein 1 and coat protein 2, respectively. The smaller protein bands that were detected by the antisera represent degraded forms of the full-size proteins (Figure 6) [25].

We did not observe any degradation products of the ORF1 (125 kDa) protein. We performed RdRp (RNA-dependent RNA polymerase) assays using [α-^32^P] UTP on MoCV1-A particles purified from 2-day-old and 14-day-old mycelia. Autoradiography of the native PAGE gel revealed radioactive signals from both types of MoCV1-A particles.

## 5. Release of Mycoviral dsRNA Genomes from the Mycelium of Mycovirus-Infected *M. oryzae* into the Culture Supernatant

In general, fungal viruses have no extracellular phase, and indeed, we have never detected virions other than the MoCV1 strains. It is believed that the extracellular phase is not required for the spread of mycovirus infection because the virus is propagated via hyphal fusion (anastomosis) between compatible individuals [7]. Hyphal fusion is limited to cases where the hyphae are compatible. In the filamentous fungus, *Podospora anserine*, the [Het-s*] phonotype, which determines compatibility for hyphal fusion, can spontaneously transition to the [Het-s] phenotype due to the prion-like action of the *het-s* gene product. As a result, mutually compatible strains suddenly become incompatible, with the development of a hyphal boundary line and programmed cell death [42]. Hyphal anastomosis seems to have the disadvantage that viruses present in one colony are readily transmitted throughout the other. To limit this problem, the process is completed only by closely related colonies that are likely to already carry the same viruses [43,44]. This can be thought of as a clever self-defence mechanism that prevents the spread of virus infection caused by hyphal fusion.

**Figure 7:**
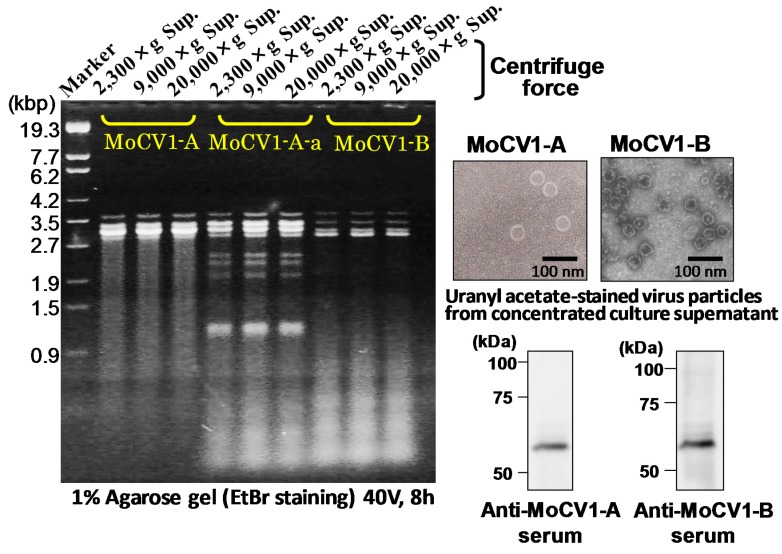
Release of mycoviruses from the mycelium of mycovirus-infected *M. oryzae* into the culture supernatant. The mycoviruses gradually appeared and accumulated in the liquid medium during the long-term suspension culture. After five weeks of liquid culture, 250 μL samples of the culture supernatant were subjected to stepwise centrifugation treatments. Total nucleic acids were extracted from each supernatant and then subjected to agarose gel electrophoresis.

We discovered that MoCV1-A is an exceptional case because the dsRNAs can exist and survive in the cell-free fraction of the liquid culture medium (Figure 7) [17]. The release of mycoviral dsRNA into the culture supernatant was also found for MoCV1-B [45]. The amount of virus-derived dsRNA detected in the culture supernatant peaked at 1 μg/mL four to five weeks after inoculation of the liquid medium. MoCV1-A and MoCV1-B viral proteins were also detected in the cell-free culture supernatant [17,18]. We investigated this phenomenon in the following additional mycoviruses: *Magnaporthe oryzae.* virus 2 (MoV2) [46], Alternaria alternata victorivirus 1 (AaVV1) [47], Alternaria alternata virus 1 (AaV1) [48], AaCV1, and L-A virus of *Saccharomyces cerevisiae* [49]. No virus-derived dsRNA was detected in the culture supernatant with any of these viruses.

The release of viruses into the liquid culture medium used for their propagation is a well-known phenomenon for animal viruses. Generally, viruses that infect animal cells reproduce rapidly and then lyse the cells so that the virions are released rapidly over a few days into the extracellular environment [50]. Conversely, MoCV1 virions are released gradually into liquid cultures in a process caused by nitrogen and carbon starvation, which takes ca. 14 days for mini-jar cultures and four to eight weeks for larger flask cultures. Examination of the protein composition of extracellular MoCV1-A virions revealed that ca. 200 amino acids at the C terminus of the ORF 3 protein had been degraded.

## 6. Heterologous Expression of Mycoviral Proteins Induced Cytological Damage in the Yeast, *Saccharomyces cerevisiae*

**Figure 8:**
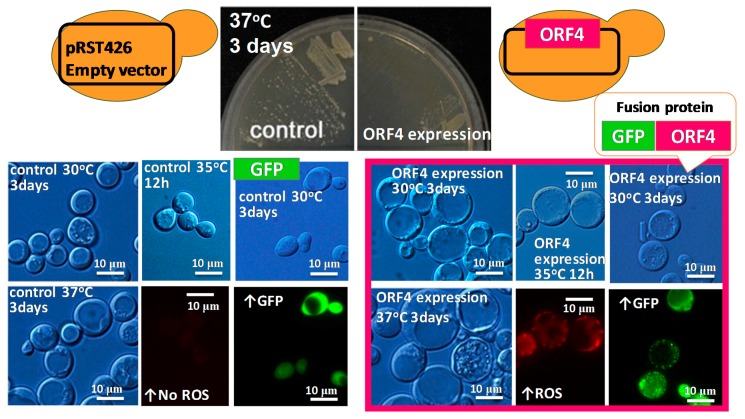
Expression of the MoCV1-A ORF4 protein induced cytological damage in yeast (*S. cerevisiae*) cells. The MoCV1-A ORF4 gene was inserted in a high-expression vector (2 μ ori, *TDH3* promoter) and the vector was used to transform the yeast strain, W303-1A. The morphology and growth of the yeast cells were observed. The MoCV1-A ORF4-GFP fusion protein caused aggregation in the yeast cells.

We attempted to use yeast (*S. cerevisiae*) to investigate the functions of the MoCV1-A encoded proteins. Of the five putative MoCV1-A proteins, ORF1 was found to have eight conserved motifs characteristic of RdRps and is assumed to function in this role. However, based on BLAST searches, we were unable to predict the functions of the other four putative proteins. Therefore, shuttle vectors were constructed by ligating each of the four MoCV1-A ORF sequences of unknown function downstream of a low expression promoter (*ADH1*) or a high expression promoter (*TDH3*) for expression in yeast cells [25]. The influence of each protein on yeast cell growth was investigated following mini jar culture in 1 L semi-synthetic media. Sampling was carried out every 2 h from 8–46 h post inoculation. Optical density, viable cell number, glucose concentration, and pH were measured together with observations of cell morphology and immunological analyses. Abnormalities in cell morphology, such as the appearance of enlarged vacuoles and vesicles, were observed when the ORF4 protein was over expressed in yeast cells (Figure 8). A series of cultivation tests revealed that ORF4 expression also caused a decrease in the rate of cell proliferation and a decrease in cell life span [38,51].

We also examined the effects of MoCV1-A ORF4 expression in the human pathogenic budding yeast, *Cryptococcus neoformans*. As with *S. cerevisiae*, over expression of the protein in *C. neoformans* caused a decrease in the growth rate, increase in emergence, and enlargement of vacuoles. Additionally, the formation of capsules, which are involved in the pathogenicity of *C. neoformans*, was also reduced in the ORF4-expressing cells, suggesting a reduction in pathogenic potential [45]. Expression of an ORF4-GFP fusion protein in *S. cerevisiae* resulted in the formation of abnormal yeast cell aggregates. In MoCV1-A, ORF4 expression also resulted in significant inhibition of growth at high temperatures (35 °C and 37 °C) as compared to cells grown at the optimal temperature (30 °C) [51] plus reduced expression of stress-response genes and increased expression of translation-related genes. We also observed the generation of reactive oxygen species in these cells (Figure 8). Further investigations on the mechanisms of cytotoxicity and growth inhibition caused by expression of the ORF4 protein in yeast and rice blast fungus cells are planned.

The MoCV1-A ORF4 protein showed significant similarity to related proteins from other viruses in cluster II of the genus, *Chrysovirus*. Multiple alignments of the ORF4-related protein sequences showed that their central regions (aa 210–591 in MoCV1-A ORF4) are relatively conserved. Indeed, yeast transformants expressing the conserved central region of the MoCV1-A ORF4 protein (325 aa–575 aa) showed similar impaired growth phenotypes to those observed in yeast expressing the full-length MoCV1-A ORF4 protein [51].

When the MoCV1-A ORF4 protein was over expressed in *E. coli*, the recombinant protein (rORF4p) was present in the insoluble fraction and when the culture reached an OD_600_ of 1.0, the yield of rORF4p was ca. 1 mg/50 mL of LB medium, which is ca. >50% of the total amount of protein in the culture. In the secretory production system utilizing *Pichia pastoris*, it is possible to produce ca. 1 mg recombinant protein per 100 mL of liquid medium [51].

## 7. Influence of MoCV1-A on the Pathogenicity of *Magnaporthe oryzae*

There are several reports showing that mycovirus infection can reduce the pathogenicity of the host fungus in plants, and this phenomenon is called hypovirulence. Hypovirulence has also been reported in the human pathogenic fungus, *A. fumigatus* [52]. In contrast, mycovirus infections sometimes confer hypervirulence to host fungi, characterized by enhanced growth and pathogenicity. Examples of hypervirulence are found in pathogens of both plants and humans [53,54]. However, few studies have examined the effects of mycoviral infection in cases of gene-for-gene interactions between plants and their fungal pathogens.

**Figure 9:**
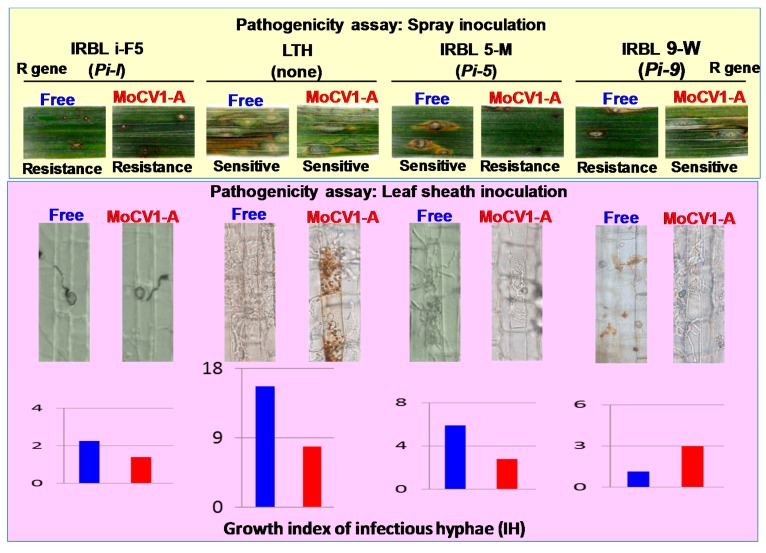
Influence of MoCV1-A on pathogenicity of *M. oryzae*. Upper panel: Spray inoculation assays revealed that MoCV1-A infection altered *M. oryzae* pathogenicity from virulence to avirulence (sensitive to resistance) in IRBL 5-M rice, and from avirulence to virulence (resistance to sensitive) in IRBL 9-W rice. Lower panel: The changes in pathogenicity were confirmed by changes in the growth indices of infectious hyphae in leaf sheath inoculation assays (lower panel). Details about the pathogenicity assays and statistical analyses are given in Aihara et al. [39].

Pathogenic races of *M. oryzae* are determined by a gene-for-gene system, where an *avirulence* gene in the pathogen induces disease resistance in a rice variety with a corresponding resistance (*R*) gene [55]. To examine whether MoCV1-A infection affects pathogenic races of *M. oryzae*, we inoculated different rice varieties with a virus-free and a MoCV1-A-infected *M. oryzae* strain. Here, inoculation of the *R* gene-free rice variety, Lijiangxintuanheigu (LTH), showed that MoCV1-A infection resulted in reduced fungal virulence and this result was supported by an analysis of invasive hyphal development on onion epidermal cells. However, when spray or leaf-sheath inoculation methods were used to inoculate monogenic rice lines carrying different *R* genes, the MoCV1-A-infected and MoCV1-A-free *M. oryzae* strains caused different lesion types (resistance to susceptible or vice versa) on individual rice varieties. These data suggest that MoCV1-A infection can alter the pathogenicity of the host *M. oryzae* from avirulence to virulence, or from virulence to avirulence, depending on the rice variety (Figure 9) [39]. These results are consistent with the frequent emergence of new pathogenic races of rice blast fungus [56]. However, we did not find any gain or loss of the fungal avirulence genes, which determine pathogenic races of the fungus.

In a recent investigation, we discovered that infection of the Japanese pear pathotype fungus, *Alternaria alternate*, with Alternaria alternata chrysovirus 1 (AaCV1) simultaneously impaired growth of the host fungus and increased levels of the host-specific AK-toxin [38,57]. This is another example of a mycovirus infection causing changes in pathogenic races of the host fungus, because *A. alternata* can infect some specific varieties of Japanese pear, but not others [58]. It is likely that the enhancement of host fungal pathogenicity in some varieties, without any mutation in the avirulence genes, is one of the strategies used by mycoviruses to survive in the agricultural ecosystem, where humans cultivate many plant varieties with different *R* genes to reduce damage by plant disease. Mycoviruses may increase survival rates by retaining the diversity of avirulence genes in their host fungi, which are important for fungal adaptation to plants [59].

## 8. Conclusions

The presence of mycoviruses infecting fungi is often detected by the appearance of morphological changes in the fungi, such as changes in their growth on agar medium. Mycoviruses that infect plant pathogenic fungi can also alter the pathogenicity of the host fungus sometimes, by changing the mode of plant infection by the fungus. We found that MoCV1-A changes the pathogenicity of its host, the rice blast fungus, *M. oryzae*. We observed growth inhibition in yeast cells due to heterologous expression of the MoCV1-A ORF4 protein. The results suggest that over expression of the ORF4 protein induces a modulation in the transcription of pathogenicity-associated genes in the host fungus. Likewise, totiviruses, partitiviruses, and chrysoviruses similar to MoCV1 are widely distributed in nature. It is possible that these mycoviruses can function as epigenetic factors that elicit changes in the pathogenicity of phytopathogenic fungi. These changes can be accompanied by alterations in the fungal host’s susceptibility to fungicides [60].
